# The Highs and Lows of Cannabis in Cancer Treatment and Bone Marrow Transplantation

**DOI:** 10.5041/RMMJ.10391

**Published:** 2020-01-30

**Authors:** Osnat Almogi-Hazan, Iman Khuja, Sivan Ritter, Reuven Or

**Affiliations:** 1 Laboratory of Immunotherapy and Bone Marrow Transplantation, Hadassah-Hebrew University Medical Center, Jerusalem, Israel; 2 Independent Contractor, Jerusalem, Israel

**Keywords:** Bone marrow transplantation, cancer, cannabidiol, cannabinoid receptor 2, cannabinoids, cannabis, endocannabinoid, immunotherapy, tumor, Δ9-tetrahydrocannabinol

## Abstract

In the last decade, we have observed an increased public and scientific interest in the clinical applications of medical cannabis. Currently, the application of cannabinoids in cancer patients is mainly due to their analgesic and anti-emetic effects. The direct effects of phyto-cannabinoids on cancer cells are under intensive research, and the data remain somewhat inconsistent. Although anti-proliferative properties were observed *in vitro*, conclusive data from animal models and clinical trials are lacking. Since immunotherapy of malignant diseases and bone marrow transplantation are integral approaches in hemato-oncology, the immuno-modulatory characteristic of cannabinoids is a fundamental aspect for consideration. The effect of cannabinoids on the immune system is presently under investigation, and some evidence for its immuno-regulatory properties has been shown. In addition, the interaction of cannabinoids and classical cytotoxic agents is a subject for further investigation. Here we discuss the current knowledge of cannabinoid-based treatments in preclinical models and the limited data in oncological patients. Particularly, we address the possible contradiction between the direct anti-tumor and the immune-modulatory effects of cannabinoids. Better understanding of the mechanism of cannabinoids influence is essential to design therapies that will allow cannabinoids to be incorporated into the clinic.

## CANNABIS AND CANCER

Cancer is one of the most prevalent and devastating diseases in the modern world. Its treatments are often highly toxic to healthy tissues, causing severe side effects. In the course of treatment, both curative and palliative care will be administered either exclusively or in combination. As a result of the disease and its curative treatments, patients experience nausea, vomiting, loss of appetite, and pain. These symptoms greatly reduce their quality of life, and first-line palliative interventions are often insufficient for symptom control.

Cannabis and cannabinoids are known for their analgesic and anti-emetic effects, and therefore their application has increased for chemotherapy-induced nausea, vomiting, and chronic pain.^1^ Whether cannabinoids have an anti-cancer affect is yet to be determined. Recent studies suggested that some cannabinoid-based treatments might have anti-tumor properties.^2^–^5^ Cannabinoids were found to modulate key cell signaling pathways involved in the control of cancer cell proliferation and survival.^6^–^8^ However, most of these studies utilized *in vitro* methods, a few were done in immune-competent animal models, and the data from human patients are anecdotal. In addition, the heterogeneity of endocannabinoids and their receptors in different tumor types raises the possibility that specific cannabinoid compositions should be used to treat differing cancer subtypes.^3^,^9^,^10^

## CANNABIS AND IMMUNITY IN CANCER PATIENTS

The tumor microenvironment is a complex ecosystem, comprising blood vessels, immune cells, fibroblasts, extracellular matrix, cytokines, hormones, and other factors. The different elements of the tumor microenvironment contribute to cancer progression. In particular, it is now evident that the immune system plays a key role in the development and progression of cancer.^11^–^13^ Immune cells possess the ability to eradicate cancer. However, in cancer patients the anti-tumor immune response is insufficient. In recent years, immunotherapy has revolutionized cancer treatment, restoring tumor-induced immune deficiency in the tumor microenvironment and modulating immune responses against cancers.^14^ Allogeneic hematopoietic stem cell transplantation (HSCT), which is usually performed for patients with hematologic malignancies, is another treatment that aims to induce anti-tumor immunity by targeting minor histocompatibility antigens on residual cancer cells. Any treatment with immune-suppressive properties may reduce the efficacy of such therapies.

The immune-regulatory properties of cannabis and cannabinoid-based treatments were demonstrated in various preclinical and clinical studies.^15^–^17^ It is therefore important to investigate the effects of cannabinoid-based treatments on the immunity of cancer patients and on the efficacy of immune-related therapies. With a greater understanding of cannabinoid-based treatment effects on the immune system we will be able to appropriately apply them to treatment of cancer patients in combination with existing therapies. Unfortunately, the basic and medical research dedicated to this subject is currently lacking.

McKallip et al. demonstrated, in a murine model of breast cancer, that the phyto-cannabinoid Δ9-tetrahydrocannabinol (THC) promotes growth of cancer cells and metastasis by suppression of the anti-tumor immune response.^18^ One group demonstrated enhanced tumor growth in THC-treated immune-competent mice but not in immune-incompetent mice in models of lung cancer,^19^ while another group showed inhibition of tumor growth by synthetic cannabinoid receptor agonists in both immune-competent mice and immune-incompetent mice, in a model of melanoma xenograft.^20^ In an *ex vivo* experiment, Zgair et al. showed that both phytocannabinoids cannabidiol (CBD) and THC have anti-proliferative effects on peripheral blood mononuclear cells (PBMC) isolated from patients on chemotherapy regimens to treat non-seminomatous germ cell tumors, which were comparable to the effect on PBMCs from healthy volunteers.^16^

Only one study has investigated the interaction between phyto-cannabinoids and immunotherapy with checkpoint inhibitors. In this retrospective, observational study in kidney cancer and melanoma patients, Taha et al. demonstrated an inverse relationship between cannabis use and the response to treatment with nivolumab, without affecting progression-free survival or overall survival and without relation to specific phyto-cannabinoid composition.^21^

## CANNABIS AND IMMUNITY IN HEMATOPOIETIC STEM CELL TRANSPLANTATION

In allogeneic HSCT the propensity of the grafted immune cells to eliminate residual tumor cells is also responsible for rejection of host tissues and the development of graft versus host disease (GVHD).^22^ In addition, slow, impaired, or dysregulated reconstitution of donor-derived immune cell populations, together with GVHD and other post-transplant complications, causes susceptibility to both common and rare infections. The early post-engraftment period is characterized by a progressive recovery of cell-mediated immunity; however, full immune reconstitution may take years.^23^

In our recently published research,^17^ we compared the consequences of treatment with THC and CBD *in vitro* and in murine bone marrow transplantation (BMT) models. Since it has been suggested that the combination of cannabinoids with other active molecules in the plant may achieve better clinical results than pure cannabinoids (known as the entourage effect),^24^ we also examined the differences between the effects of the pure cannabinoids and high THC/high CBD cannabis extracts. Cannabis extracts with a high content (20%–30%) of CBD or THC were named CBD botanical drug substance (BDS) or THC BDS, respectively.

**Figure 1:**
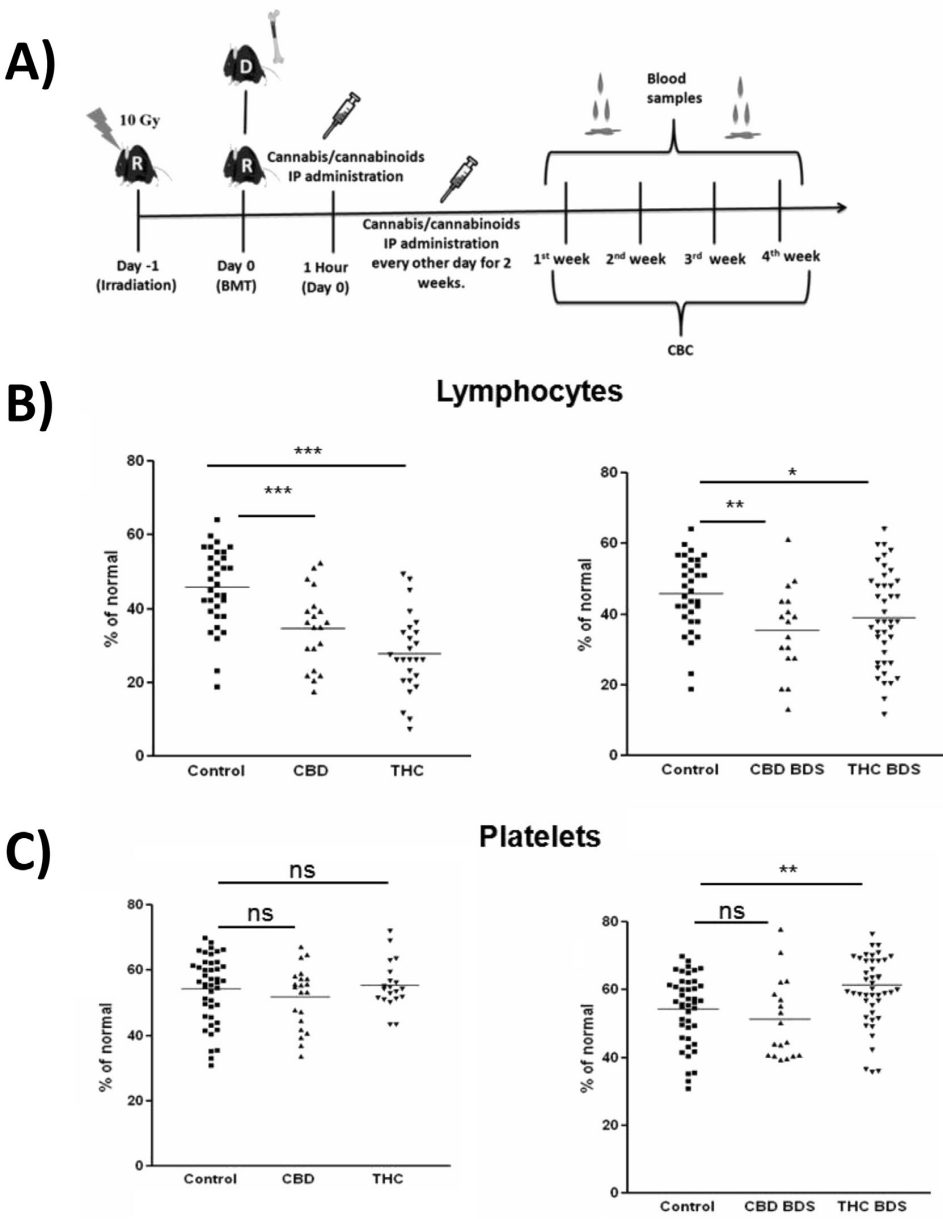
Cannabis/Cannabinoids Administration to Syngeneic Bone Marrow Transplantation Model. A: Recipient C57BL/6 mice (R) received lethal whole-body irradiation and were reconstituted with 8×10^6^ donor C57BL/6 (D) bone marrow cells. Cannabis/cannabinoids were administered IP every other day, for 2 weeks from the day of transplantation. Blood samples for complete blood counts were obtained once a week. B: Lymphocyte counts (day 21 after transplantation) in pure cannabinoid-treated groups (left) and BDS-treated groups (right) are presented. C: Platelet counts in pure cannabinoid-treated groups (left) and BDS-treated groups (right), day 14 after transplantation. * *P*<0.05; ** *P*<0.001; *** *P*<0.0001. Modified from Figure 4 in Khuja et al.^17^; reused under creative commons license (CC BY 4.0). BDS, botanical drug substance; BMT, bone marrow transplantation; IP, intraperitoneally; THC, Δ9-tetrahydrocannabinol.

**Figure 2:**
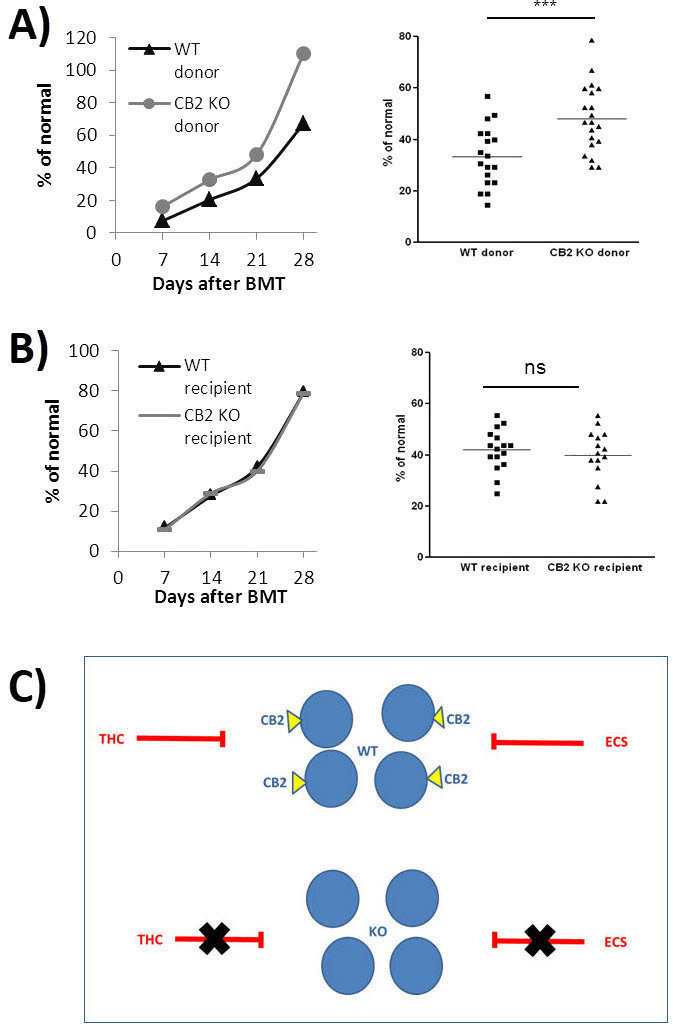
The Role of CB2 in Lymphocyte Recovery. **A:** Syngeneic BMT from CB2 KO donor mice to C57BL/6 WT mice. Average lymphocyte counts at different time points (left) and counts on day 21 after transplantation (right). *** *P*<0.0001. **B:** Syngeneic BMT from C57BL/6 WT donor mice to CB2 KO mice. Average lymphocyte counts at different time points (left) and counts on day 21 after transplantation (right). **C:** Model: CB2 has an inhibitory effect on post-transplant rehabilitation of blood lymphocytes. The WT graft rehabilitation will be delayed by endo- (ECS) and/or phyto-cannabinoid (THC) signaling (red T-bars) through CB2. Since the knockout graft does not express CB2, this inhibition is removed. Modified from Figure 5 in Khuja et al.^17^; reused under creative commons license (CC BY 4.0). BMT, bone marrow transplantation; ECS, endocannabinoids; CB2 KO, CB2 knockout; THC, Δ9-tetrahydrocannabinol; WT, wild type.

To investigate the effect of THC, CBD, and cannabis extracts on hematopoiesis after BMT *in vivo*, we utilized a syngeneic transplantation model (Figure 1A). Mice underwent lethal whole-body irradiation and were reconstituted with donor bone marrow cells. The cannabinoid treatments were administered intraperitoneally (IP) from the day of transplantation, every other day, for 2 weeks. Once a week, blood was collected for complete blood counts. Surprisingly, all treatments—and especially THC—inhibited lymphocyte reconstitution after transplantation (Figure 1B). Only the high-THC extract improved platelet rehabilitation (Figure 1C). Indeed, using knockout mice as donors, we have demonstrated that the cannabinoid receptor 2 (CB2), known to be activated by THC, has an inhibitory effect on post-transplant recovery of blood lymphocytes (Figure 2).

**Figure 3:**
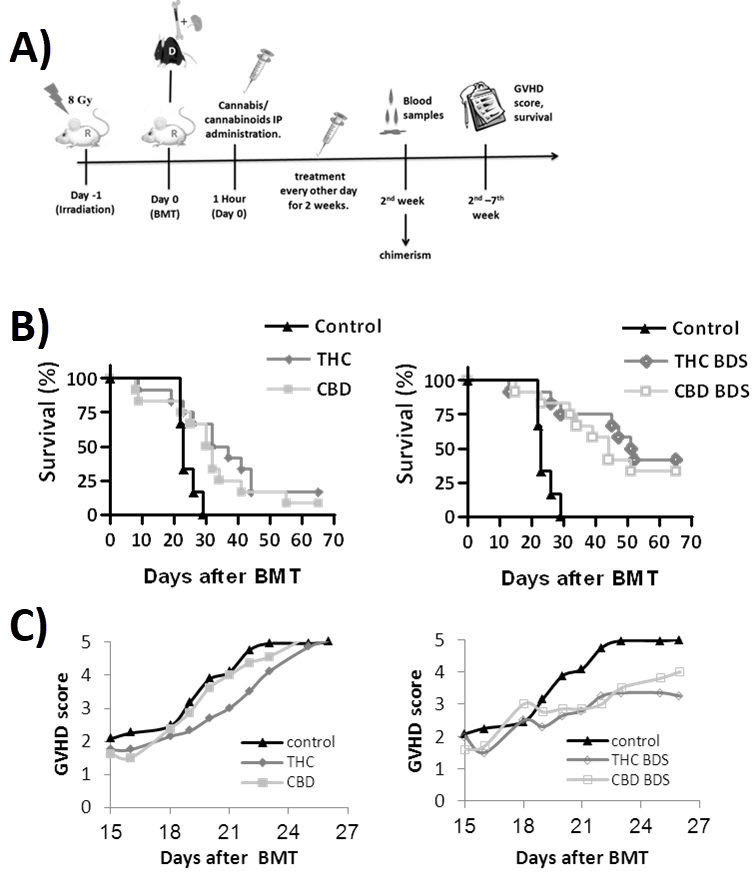
Cannabis/Cannabinoids Administration for Graft versus Host Disease (GVHD) Prophylaxis. **A:** Recipient BALB/c mice (R) received lethal whole-body irradiation and were reconstituted with 8×10^6^ donor C57BL/6 (D) bone marrow cells and 2×10^6^ spleen cells. Cannabis/cannabinoids were administered IP every other day, for 2 weeks from the day of transplantation. The clinical condition of the mice was evaluated for up to 67 days after transplantation. **B:** Survival curve THC and CBD, pure cannabinoids treated groups; THC BDS and CBD BDS, cannabis extracts treated groups. **C:** Average GVHD score THC and CBD, pure cannabinoids treated groups; THC BDS and CBD BDS, cannabis extracts treated groups. Modified from Figure 6 in Khuja et al.^17^; reused under creative commons license (CC BY 4.0). BDS, botanical drug substance; CBD, cannabidiol; IP, intraperitoneally; TCH, Δ9-tetrahydrocannabinol.

Since cannabinoids have anti-inflammatory functions, we used an allogeneic transplantation model to compare the potential influences of the cannabinoid treatments in the prevention of GVHD. Although pure cannabinoids had a superior effect in our *in vitro* studies, cannabis extracts were better than pure cannabinoids at reducing the severity of disease and improving survival in the GVHD model (Figure 3).

These results highlight the complexity of using cannabinoid-based drugs and the need for additional comparative scientific research. In agreement with our results, in a clinical trial Yeshurun et al. showed that in CBD-treated groups there was a low incidence of acute GVHD, and a reduced rate of moderate and severe chronic GVHD was demonstrated compared to historical controls.^25^

## CONCLUSIONS

To conclude, cannabinoid-based treatments have beneficial palliative properties in oncological patients and may have anti-tumor effects in specific cancer subtypes. However, better understanding of cannabinoid direct anti-tumor effects, and its influence on the immune system, is essential for the integration of cannabinoids into the clinician’s armamentarium.

## Abbreviations

BMT: bone marrow transplantation
BDS: botanical drug substance
CBD: cannabidiol
CB2: cannabinoid receptor 2
THC: Δ9-tetrahydrocannabinol
GVHD: graft versus host disease
HSCT: hematopoietic stem cell transplantation
IP: intraperitoneally
KO: knockout
PBMC: peripheral blood mononuclear cells
WT: wild type.
